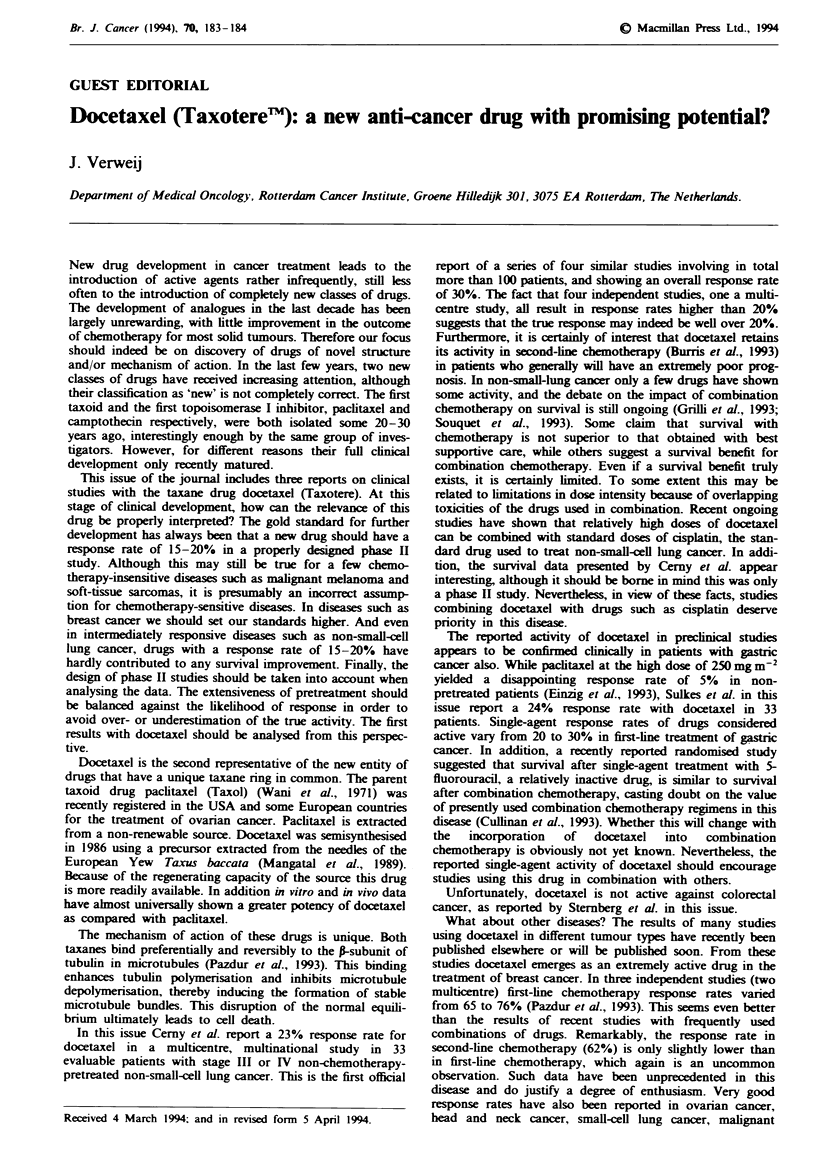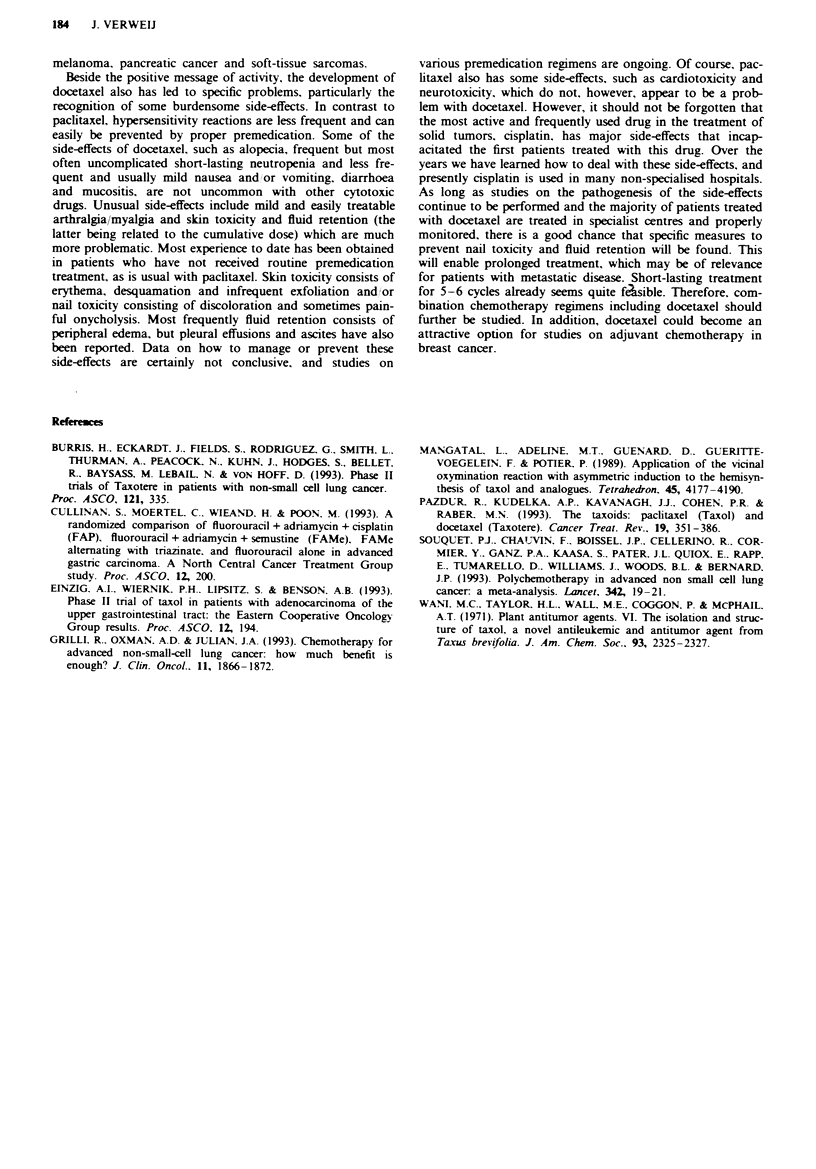# Docetaxel (Taxotere): a new anti-cancer drug with promising potential?

**DOI:** 10.1038/bjc.1994.276

**Published:** 1994-08

**Authors:** J. Verweij


Br. J. Cancer (1994), 70, 183-184 © Macmillan Press Ltd., 1994

GUEST EDITORIAL

### Docetaxel (Taxotere™): a new anti-cancer drug with promising potential?

J. Verweij

*Department of Medical Oncology, Rotterdam Cancer Institute, Groene Hilledijk 301, 3075 EA Rotterdam, The Netherlands.*

New drug development in cancer treatment leads to the introduction of active agents rather infrequently, still less often to the introduction of completely new classes of drugs. The development of analogues in the last decade has been largely unrewarding, with little improvement in the outcome of chemotherapy for most solid tumours. Therefore our focus should indeed be on discovery of drugs of novel structure and/or mechanism of action. In the last few years, two new classes of drugs have received increasing attention, although their classification as 'new' is not completely correct. The first taxoid and the first topoisomerase I inhibitor, paclitaxel and camptothecin respectively, were both isolated some 20-30 years ago, interestingly enough by the same group of investigators. However, for different reasons their full clinical development only recently matured.

This issue of the journal includes three reports on clinical studies with the taxane drug docetaxel (Taxotere). At this stage of clinical development, how can the relevance of this drug be properly interpreted? The gold standard for further development has always been that a new drug should have a response rate of 15-20% in a properly designed phase II study. Although this may still be true for a few chemotherapy-insensitive diseases such as malignant melanoma and soft-tissue sarcomas, it is presumably an incorrect assumption for chemotherapy-sensitive diseases. In diseases such as breast cancer we should set our standards higher. And even in intermediately responsive diseases such as non-small-cell lung cancer, drugs with a response rate of 15-20% have hardly contributed to any survival improvement. Finally, the design of phase II studies should be taken into account when analysing the data. The extensiveness of pretreatment should be balanced against the likelihood of response in order to avoid over- or underestimation of the true activity. The first results with docetaxel should be analysed from this perspective.

Docetaxel is the second representative of the new entity of drugs that have a unique taxane ring in common. The parent taxoid drug paclitaxel (Taxol) (Wani *et al.*, 1971) was recently registered in the USA and some European countries for the treatment of ovarian cancer. Paclitaxel is extracted from a non-renewable source. Docetaxel was semisynthesised in 1986 using a precursor extracted from the needles of the European Yew *Taxus baccata* (Mangatal *et al.*, 1989). Because of the regenerating capacity of the source this drug is more readily available. In addition *in vitro* and *in vivo* data have almost universally shown a greater potency of docetaxel as compared with paclitaxel.

The mechanism of action of these drugs is unique. Both taxanes bind preferentially and reversibly to the β-subunit of tubulin in microtubules (Pazdur *et al.*, 1993). This binding enhances tubulin polymerisation and inhibits microtubule depolymerisation, thereby inducing the formation of stable microtubule bundles. This disruption of the normal equilibrium ultimately leads to cell death.

In this issue Cerny *et al.* report a 23% response rate for docetaxel in a multicentre, multinational study in 33 evaluable patients with stage III or IV non-chemotherapy-pretreated non-small-cell lung cancer. This is the first official report of a series of four similar studies involving in total more than 100 patients, and showing an overall response rate of 30%. The fact that four independent studies, one a multicentre study, all result in response rates higher than 20% suggests that the true response may indeed be well over 20%. Furthermore, it is certainly of interest that docetaxel retains its activity in second-line chemotherapy (Burris *et al.*, 1993) in patients who generally will have an extremely poor prognosis. In non-small-lung cancer only a few drugs have shown some activity, and the debate on the impact of combination chemotherapy on survival is still ongoing (Grilli *et al.*, 1993; Souquet *et al.*, 1993). Some claim that survival with chemotherapy is not superior to that obtained with best supportive care, while others suggest a survival benefit for combination chemotherapy. Even if a survival benefit truly exists, it is certainly limited. To some extent this may be related to limitations in dose intensity because of overlapping toxicities of the drugs used in combination. Recent ongoing studies have shown that relatively high doses of docetaxel can be combined with standard doses of cisplatin, the standard drug used to treat non-small-cell lung cancer. In addition, the survival data presented by Cerny *et al.* appear interesting, although it should be borne in mind this was only a phase II study. Nevertheless, in view of these facts, studies combining docetaxel with drugs such as cisplatin deserve priority in this disease.

The reported activity of docetaxel in preclinical studies appears to be confirmed clinically in patients with gastric cancer also. While paclitaxel at the high dose of 250 mg $m^{-2}$ yielded a disappointing response rate of 5% in non-pretreated patients (Einzig *et al.*, 1993), Sulkes *et al.* in this issue report a 24% response rate with docetaxel in 33 patients. Single-agent response rates of drugs considered active vary from 20 to 30% in first-line treatment of gastric cancer. In addition, a recently reported randomised study suggested that survival after single-agent treatment with 5-fluorouracil, a relatively inactive drug, is similar to survival after combination chemotherapy, casting doubt on the value of presently used combination chemotherapy regimens in this disease (Cullinan *et al.*, 1993). Whether this will change with the incorporation of docetaxel into combination chemotherapy is obviously not yet known. Nevertheless, the reported single-agent activity of docetaxel should encourage studies using this drug in combination with others.

Unfortunately, docetaxel is not active against colorectal cancer, as reported by Sternberg *et al.* in this issue.

What about other diseases? The results of many studies using docetaxel in different tumour types have recently been published elsewhere or will be published soon. From these studies docetaxel emerges as an extremely active drug in the treatment of breast cancer. In three independent studies (two multicentre) first-line chemotherapy response rates varied from 65 to 76% (Pazdur *et al.*, 1993). This seems even better than the results of recent studies with frequently used combinations of drugs. Remarkably, the response rate in second-line chemotherapy (62%) is only slightly lower than in first-line chemotherapy, which again is an uncommon observation. Such data have been unprecedented in this disease and do justify a degree of enthusiasm. Very good response rates have also been reported in ovarian cancer, head and neck cancer, small-cell lung cancer, malignant

Received 4 March 1994; and in revised form 5 April 1994.

melanoma, pancreatic cancer and soft-tissue sarcomas.

Beside the positive message of activity, the development of docetaxel also has led to specific problems, particularly the recognition of some burdensome side-effects. In contrast to paclitaxel, hypersensitivity reactions are less frequent and can easily be prevented by proper premedication. Some of the side-effects of docetaxel, such as alopecia, frequent but most often uncomplicated short-lasting neutropenia and less frequent and usually mild nausea and/or vomiting, diarrhoea and mucositis, are not uncommon with other cytotoxic drugs. Unusual side-effects include mild and easily treatable arthralgia/myalgia and skin toxicity and fluid retention (the latter being related to the cumulative dose) which are much more problematic. Most experience to date has been obtained in patients who have not received routine premedication treatment, as is usual with paclitaxel. Skin toxicity consists of erythema, desquamation and infrequent exfoliation and/or nail toxicity consisting of discoloration and sometimes painful onycholysis. Most frequently fluid retention consists of peripheral edema, but pleural effusions and ascites have also been reported. Data on how to manage or prevent these side-effects are certainly not conclusive, and studies on various premedication regimens are ongoing. Of course, paclitaxel also has some side-effects, such as cardiotoxicity and neurotoxicity, which do not, however, appear to be a problem with docetaxel. However, it should not be forgotten that the most active and frequently used drug in the treatment of solid tumors, cisplatin, has major side-effects that incapacitated the first patients treated with this drug. Over the years we have learned how to deal with these side-effects, and presently cisplatin is used in many non-specialised hospitals. As long as studies on the pathogenesis of the side-effects continue to be performed and the majority of patients treated with docetaxel are treated in specialist centres and properly monitored, there is a good chance that specific measures to prevent nail toxicity and fluid retention will be found. This will enable prolonged treatment, which may be of relevance for patients with metastatic disease. Short-lasting treatment for 5–6 cycles already seems quite feasible. Therefore, combination chemotherapy regimens including docetaxel should further be studied. In addition, docetaxel could become an attractive option for studies on adjuvant chemotherapy in breast cancer.

### References

BURRIS, H., ECKARDT, J., FIELDS, S., RODRIGUEZ, G., SMITH, L., THURMAN, A., PEACOCK, N., KUHN, J., HODGES, S., BELLET, R., BAYSASS, M. LEBAIL, N. & VON HOFF, D. (1993). Phase II trials of Taxotere in patients with non-small cell lung cancer. *Proc. ASCO*, **121**, 335.

CULLINAN, S., MOERTEL, C., WIEAND, H. & POON, M. (1993). A randomized comparison of fluorouracil + adriamycin + cisplatin (FAP), fluorouracil + adriamycin + semustine (FAMe), FAMe alternating with triazinate, and fluorouracil alone in advanced gastric carcinoma. A North Central Cancer Treatment Group study. *Proc. ASCO*, **12**, 200.

EINZIG, A.I., WIERNIK, P.H., LIPSITZ, S. & BENSON, A.B. (1993). Phase II trial of taxol in patients with adenocarcinoma of the upper gastrointestinal tract: the Eastern Cooperative Oncology Group results. *Proc. ASCO*, **12**, 194.

GRILLI, R., OXMAN, A.D. & JULIAN, J.A. (1993). Chemotherapy for advanced non-small-cell lung cancer: how much benefit is enough? *J. Clin. Oncol.*, **11**, 1866–1872.

MANGATAL, L., ADELINE, M.T., GUENARD, D., GUERITTE-VOEGELEIN, F. & POTIER, P. (1989). Application of the vicinal oxymination reaction with asymmetric induction to the hemisynthesis of taxol and analogues. *Tetrahedron*, **45**, 4177–4190.

PAZDUR, R., KUDELKA, A.P., KAVANAGH, J.J., COHEN, P.R. & RABER, M.N. (1993). The taxoids: paclitaxel (Taxol) and docetaxel (Taxotere). *Cancer Treat. Rev.*, **19**, 351–386.

SOUQUET, P.J., CHAUVIN, F., BOISSEL, J.P., CELLERINO, R., CORMIER, Y., GANZ, P.A., KAASA, S., PATER, J.L. QUIOX, E., RAPP, E., TUMARELLO, D., WILLIAMS, J., WOODS, B.L. & BERNARD, J.P. (1993). Polychemotherapy in advanced non small cell lung cancer: a meta-analysis. *Lancet*, **342**, 19–21.

WANI, M.C., TAYLOR, H.L., WALL, M.E., COGGON, P. & MCPHAIL, A.T. (1971). Plant antitumor agents. VI. The isolation and structure of taxol, a novel antileukemic and antitumor agent from *Taxus brevifolia*. *J. Am. Chem. Soc.*, **93**, 2325–2327.